# Secondary infections in the intensive care unit do not worsen long-term functional outcomes in sepsis survivors

**DOI:** 10.62675/2965-2774.20260247

**Published:** 2026-02-20

**Authors:** Taciana de Castilhos Cavalcanti, Miriane Melo Silveira Moretti, Cassiano Teixeira, Daniel Sganzerla, Regis Goulart Rosa, Karina de Oliveira Azzolin

**Affiliations:** 1 Universidade Federal do Rio Grande do Sul Hospital de Clínicas de Porto Alegre Porto Alegre RS Brazil Intensive Care Unit, Hospital de Clínicas de Porto Alegre, Universidade Federal do Rio Grande do Sul - Porto Alegre (RS), Brazil.; 2 Inova Medical Research - Health Care Porto Alegre RS Brazil Inova Medical Research - Health Care - Porto Alegre (RS), Brazil.; 3 Hospital Moinhos de Vento Department of Internal Medicine Porto Alegre RS Brazil Department of Internal Medicine, Hospital Moinhos de Vento - Porto Alegre (RS), Brazil.

Sepsis often triggers a cascade of immunosuppressive events that can leave patients vulnerable to secondary infections during their intensive care unit (ICU) stay.^(1)^ These infections - most commonly pneumonia, bloodstream, and urinary tract infections - are thought to worsen both short- and long-term outcomes. However, whether ICU-acquired infections contribute to persistent functional disability in survivors of sepsis remains unclear.

This is a secondary analysis of a prospective multicenter cohort study conducted in 10 Brazilian tertiary hospitals to investigate the long-term consequences of ICU-acquired secondary infections on functional capacity.^(1)^ From May 2016 to December 2018, we enrolled adult patients admitted with sepsis who survived at least 72 hours in the ICU. The primary outcome was a decline in functional capacity at 6 months post-discharge, measured using the Barthel Index. Secondary infections were defined by Centers for Disease Control and Prevention (CDC) criteria and included pneumonia, bloodstream infection, and urinary tract infection acquired ≥ 48 hours after ICU admission. We defined sepsis according to the Sepsis-3 consensus criteria.^(2)^

Of the 522 patients enrolled, 95 (18.2%) acquired a secondary infection - most commonly pneumonia (15.1%). Compared to those without secondary infections, affected patients were more likely to require mechanical ventilation (93.7% *versus* 58.5%), vasopressors (93.7% *versus* 74.7%), and renal replacement therapy (38.9% *versus* 16.2%), all p < 0.001. Intensive care unit and hospital length of stay were significantly more prolonged in those with secondary infections (median ICU stay: 21 *versus* 7 days; p < 0.001). Despite greater illness severity and higher resource utilization during the ICU stay, as indicated by increased need for organ support, long-term functional outcomes did not differ after adjusting for age, Charlson comorbidity index, and number of organ dysfunctions. A reduction in physical function was observed in 54.2% of patients with secondary infections and 60.5% of those without (hazard ratio [HR] 1.02; 95% CI 0.84 - 1.24; p = 0.45). Rates of physical dependence, readmission, and cumulative 6-month mortality were also similar between groups (Table 1).

**Table 1:** Intensive care unit characteristics and 6-month outcomes in sepsis survivors according to intensive care unit-acquired infection

Characteristic	With ICU infection (n = 95)	Without ICU infection (n = 427)	Hazard ratio (95%CI)	p value
Age (years)	62 (41.5 - 70.5)	65 (50 - 77)	-	0.01
Charlson comorbidity index	2 (1 - 4)	2 (1 - 4)	-	0.75
Number of organ dysfunctions	3 (2 - 4)	2 (1 - 3)	-	< 0.001
Need for mechanical ventilation	89 (93.7)	250 (58.5)	-	< 0.001
Need for a vasopressor	89 (93.7)	319 (74.7)	-	< 0.001
Need for renal replacement therapy	37 (38.9)	69 (16.2)	-	< 0.001
Physical dependence (%)	18 (30.5)	94 (32.9)	0.94 (0.61 - 1.45)	0.84
Decrease in physical functionality*	32 (54.2)	173 (60.5)	1.02 (0.84 - 1.24)	0.45
Cumulative rehospitalization	47 (50.5)	210 (51.9)	0.91	-
Cumulative mortality	26 (27.4)	106 (24.8)	1.08 (0.70 - 1.68)	0.72

ICU - intensive care unit.

* Physical dependence is defined as a Barthel Index ≤ 75. Hazard ratio adjusted for age, comorbidities, illness severity, and need for organ support during intensive care unit stay. Results expressed as median (interquartile range) or n (%).

Our findings suggest that while secondary infections clearly increase resource use and in-hospital morbidity, they do not appear to contribute to post-ICU functional decline in sepsis survivors. This supports prior data indicating that long-term impairment after sepsis is multifactorial and may not be driven by nosocomial complications alone.^(3-5)^ Sepsis itself induces persistent immune dysregulation and musculoskeletal deconditioning, which are likely the primary contributors to post-intensive care syndrome.^(6-10)^ Strengths of our study include its prospective design, large sample size, and multicenter scope, which improve generalizability. Limitations include reliance on a single functional measure (the Barthel Index) and a lack of detailed data on infection severity and treatment. Moreover, patients who died before hospital discharge were excluded, potentially underestimating the actual burden of secondary infections. The Barthel Index was administered by trained research personnel via structured interviews, either in person or by telephone. We acknowledge that Barthel Index scores may also be influenced by unmeasured cognitive dysfunction, which was not assessed in this study.

In conclusion, ICU-acquired infections in septic patients are markers of acute severity but do not seem to impact long-term physical recovery. Preventing these infections remains a clinical priority given their association with prolonged organ support and ICU stay. However, rehabilitation strategies targeting sepsis-induced weakness and disability may have a greater role in improving survivors’ functional trajectories.

## Data Availability

The contents underlying the research text are included in the manuscript
